# Hysteroscopic Identification of Intrauterine Pathology in Oocyte Donation Cycles: A Retrospective Study

**DOI:** 10.7759/cureus.37470

**Published:** 2023-04-12

**Authors:** Nikolaos Peitsidis, Ioannis Tsakiridis, Robert Najdecki, Georgios Michos, Foteini Chouliara, Fotios Zachomitros, Ioannis Kalogiannidis, Apostolos Athanasiadis, Evangelos Papanikolaou

**Affiliations:** 1 Private In Vitro Fertilization (IVF) Unit, Assisting Nature Centre of Reproduction and Genetics, Thessaloniki, GRC; 2 Third Department of Obstetrics and Gynaecology, School of Medicine, Faculty of Health Sciences, Aristotle University of Thessaloniki, Thessaloniki, GRC

**Keywords:** ivf, oocyte recipients, adhesions, submucous fibroids, congenital uterine anomalies, polyp, intrauterine pathology, hysteroscopy

## Abstract

Background: Hysteroscopy remains the gold standard for the diagnosis and treatment of intracavitary uterine anomalies. As for recipients where oocyte donation is mandatory, accurate evaluation of previously missed intrauterine pathology may be an important step to optimize implantation process. The aim of this study was to hysteroscopically assess the incidence of unidentified intrauterine pathology prior to embryo transfer in an oocyte recipient population.

Methods: A retrospective descriptive study was conducted between 2013 and 2022 at Assisting Nature In Vitro Fertilization (IVF) Centre in Thessaloniki, Greece. The study population consisted of oocyte recipient women who underwent hysteroscopy one-three months before embryo transfer. Furthermore, oocyte recipients after repeated implantation failure were investigated as a subgroup. Any identified pathology was treated accordingly.

Results: In total, 180 women underwent diagnostic hysteroscopy prior to embryo transfer with donor oocytes. The mean maternal age at the time of intervention was 38.9 (+5.2) years, while the mean duration of infertility was 6.03 (+1.23) years. Additionally, 21.7% (n=39) of the study population had abnormal hysteroscopic findings. In particular, congenital uterine anomalies (U1a: 1.1% {n=2}, U2a: 5.6% {n=10}, U2b: 2.2% {n=4}) and polyps (n=16) were the main findings in the sample population. Furthermore, 2.8% (n=5) had submucous fibroids and 1.1% (n=2) were diagnosed with intrauterine adhesions. Notably, in recipients after repeated implantation failure intrauterine pathology rates were even higher (39.5%).

Conclusions: Oocyte recipients and especially those with repeated implantation failures probably have high rates of previously undiagnosed intrauterine pathology so, hysteroscopy would be justified in these subfertile populations.

## Introduction

Hysteroscopy is considered the best method for visualization of the uterine cavity and investigation of intrauterine pathology; it can be done under general or local anesthesia, while outpatient diagnostic hysteroscopy using a vaginoscopic (“no touch”) technique is always an option, especially in the initial investigation of infertile patients [1,2]. Published data comparing ultrasound, saline infusion sonography and hysteroscopy for the diagnosis of endometrial pathology, reported sensitivity of 89%, 91.8% and 97.3%, respectively, thus favoring hysteroscopy [3]. Furthermore, hysteroscopy successfully integrates clinical practice into “see and treat” modality providing more accurate diagnostic preciseness [4].

The number of infertile couples is growing worldwide and despite technological advances, live birth rates in women undergoing in vitro fertilization (IVF) is still about 29% [5]. Moreover, even after pre-implantation genetic screening, pregnancy rates do not exceed 64%, which means that other maternal factors may play a key role in the success of an IVF cycle [6]. Uterine factors, such as congenital anomalies, leiomyomas, adhesions, or molecular alterations, may negatively impact the implantation of an embryo [7]. Diagnostic-operative hysteroscopy is nowadays regarded as the gold standard technique for the management of the aforementioned intrauterine abnormalities that could potentially impact fertility [8].

The societal trend towards delaying motherhood in the developed countries is associated with a high decline in the natural reproductive potential; for this reason, such couples increasingly find the oocyte receipt necessary to achieve pregnancy [9]. Similarly, oocyte donation is suggested for a subgroup of patients who have failed many times with their own eggs and are considered poor implanters [10]. The efficacy of oocyte donation is the highest of all assisted reproduction treatments because the donors are young and have no history of health issues, but it still does not exceed delivery rates of about 50%, meaning that other maternal factors might still exist [11]. The present study aimed to assess the incidence of obscured intrauterine pathology, unidentified through the previous infertility history, in oocyte recipients.

## Materials and methods

This retrospective descriptive study included oocyte recipients of Caucasian origin that underwent hysteroscopy one-three months prior to embryo transfer (between 2013 and 2022) in Assisting Nature Centre Reproduction and Genetics, IVF Unit, Thessaloniki, Greece. All women had primary infertility due to various factors. Exclusion criteria were: i) history of hysteroscopy in the past six months and ii) current sonographic diagnosis of intrauterine pathology.

All the recipients underwent routine evaluation during their early follicular phase, one-three months before the start of a new hormone replacement therapy cycle with donor oocytes. Moreover, patients planned for hysteroscopy were offered contraceptive pill (drospirenone and ethinylestradiol or chlormadinone and ethinylestradiol) started on cycle day-3, in order to achieve better cavity visualization. A vaginoscopic approach hysteroscopy was performed from day 6 to 13 of menstrual cycle under sedation. Briefly, a rigid hysteroscope -Storz Bettochi 4.8mm hysteroscope (continuous flow; 30° forward oblique view; KARL STORZ Endoscopy, USA) using 0.9% normal saline was used. Following adequate distension of the uterine cavity, systematic inspection was performed. Any intervention during hysteroscopy was performed by the Wolf endoscopic scissor 2mm and micro-forceps. In patients with submucous myomas, Figo type 0 or 1, removal of the lesions was achieved using monopolar diathermy through cutting loops and glycine as distending medium. All women were prescribed a four-day postoperative course of prophylactic oral antibiotics (doxycycline, 100mg BD) to prevent possible intrauterine infections. Notably, two senior reproductive consultants (R.N. and E.P.) performed all the procedures to prevent the possible confounding factor of multiple operators (inter-observer variability) that may adversely impact the evaluation of intrauterine view and outcomes.

Special consideration was given to a subgroup population with repeated implantation failures leading to donor oocyte receipt (RIF-DOR); in our sample, it was defined as women having more than six blastocysts transferred unsuccessfully through previous IVF cycles with their own eggs. 

Statistical analysis

Qualitative variables are described as frequencies (n) and percentages (%), while the quantitative variables as mean (Standard deviation - SD). The Chi-square test was employed to assess the differences between the total population and the RIF-DOR. Statistical significance was considered p value <0.05. The statistical program SPSS, Version 25.0 (IBM Corp., Armonk, NY) was used for calculations.

Ethics

The study was approved by the Institutional Review Board of Assisting Nature, Center of Assisted Reproduction and Genetics, Thessaloniki, Greece (Registration Number: 0501201404). Additional informed consent was obtained from all the participants.

## Results

Hysteroscopic operations were performed in 180 oocyte recipients. The mean maternal age was 38.9 (+5.2) years with the mean duration of infertility of 6.03 (+1.23) years. Patients' demographic data are presented in Table 1. The reasons for oocyte donation were advanced maternal age (>40 years, 70%), repeated implantation failure in 21%, premature menopause in 7% and history of recurrent pregnancy loss 2%. Of note, 28% of them had a history of a previous hysteroscopy.

**Table 1:** Demographic characteristics of the oocyte recipients (n=180)

Description	Hysteroscopy Group (n=180)
Age (years ± SD)	38.9 (± 5.2)
Patients with history of previous hysteroscopy n (%)	50 (28%)
Duration of infertility (years)	6.03 (± 1.23)
Reason for oocyte donation	
Advanced maternal age	126 (70%)
Repeated implantation failure	38 (21%)
Premature menopause	2 (7%)
Recurrent pregnancy loss	4 (2%)

In 78.3% (n=141), a hysteroscopy revealed normal intrauterine cavity, while 21.7% (n=39) of the participants had an underlying intrauterine pathology (Table 2). In the RIF-DOR group, which included 38 women, we identified a higher incidence of uterine anomalies compared to the rest of our sample. Particularly, there were pathological findings in 15 women of the RIF-DOR group, which was significantly higher than the total study population (39.5 vs 21.7%; p=0.006) (Table 3). 

**Table 2:** Distribution of hysteroscopic findings in oocyte recipients U1a: T shape uterus; U2a: partial septate, arcuate uterus; U2b: septate uterus

Diagnosis	Cases	Percentage
Normal Uterus	141	78.3%
Polyps	16	8.9%
U1a	2	1.1%
U2a	10	5.6%
U2b	4	2.2%
Submucous Fibroids	5	2.8%
Adhesions	2	1.1%
Total	180	100%

**Table 3:** Intrauterine pathology findings in the subgroup of oocyte recipients with previous history of repeated implantation failures. RIF-DOR:  Repeated implantation failures leading to donor oocyte receipt; U1a: T shape uterus; U2a: partial septate, arcuate uterus; U2b: septate uterus

Diagnosis	Cases	Percentage
Normal Uterus	23	60.5%
Polyps	5	13.1%
U1a	0	0%
U2a	4	10.5%
U2b	2	5.3%
Submucous Fibroids	2	5.3%
Adhesions	2	5.3%
Total	38	100%

Of note, there were 2 (1.1%) minor complications related to the hysteroscopic procedure: one during cervix dilatation, initial false route that was corrected under ultrasound guidance and one during operative procedure; moderate bleeding continued after septum resection and patient required six hours of close monitoring before discharge. Both were diagnosed at the time of surgery and managed accordingly without further complications.

## Discussion

According to the findings of the present study, one out of five donor oocyte recipients may be diagnosed with intrauterine pathology, while about two out of five RIF-DOR could have abnormal hysteroscopic findings before embryo transfer.

The bottleneck of IVF treatment is the embryo implantation; in malformations involving the endometrial cavity, implantation is disturbed and pregnancy rates could be decreased [12]. Hysteroscopy is the gold standard for early diagnosis and correction of intrauterine pathology, if present, aiming to improve pregnancy rates [13]. Despite that, there is not enough scientific evidence available on the intrauterine cavity evaluation during hysteroscopy, especially involving oocyte recipients. Of note, although the introduction of three-dimensional ultrasound has more potential than the transvaginal scan and saline infusion sonography, it still has a reduced sensitivity and specificity, compared to hysteroscopy (68.2% and 91.5%, respectively) [14].

Observational data have reported higher pregnancy rates after the hysteroscopic removal of endometrial polyps, submucous fibroids, uterine septum, or intrauterine adhesions in women seeking treatment for subfertility [15]. In our study population, which exclusively consisted of oocyte recipients, the intrauterine abnormal findings were significantly higher (21.7%) than the reported 10-15% in subfertile women undergoing IVF [8]. Hence, even if the efficacy of oocyte donation is the highest of all assisted reproduction treatments [16], our findings may strengthen the role of pre-embryo transfer hysteroscopic evaluation of these women.

With regards to the RIF-DOR group, we identified intrauterine pathology in the 39.5% of the participants, which is in accordance with previously published data concluding that patients with a history of RIF should probably undergo hysteroscopic examination before any further cycles are considered [17].

As for the study limitations, its retrospective design and the limited number of patients should be reported. These factors are well known variables that may influence the accuracy of findings. Furthermore, this is a single-center study, so the results should not be generalized for the total population. On the other hand, the main strength of this study is mainly that, all subjects were oocyte recipients, a concrete population on which no previous similar studies were conducted. Furthermore, all the hysteroscopic operations were performed by the same two IVF specialized endoscopists, thus, minimizing the possible inter-observer variability.

The results of this study indicate that oocyte recipients have uterine abnormalities which could not be diagnosed by other available techniques and that hysteroscopy could be a valuable tool in the management of infertility. Hysteroscopy has proved its value and has now become a usual easy applicable and carried out as an outpatient procedure [4]. Of note, the procedure itself is not without complications [17]. In this study complication rates were about 1% and all of them resolved easily; this is in accordance with the reported incidence of complications of about 1-3% [17].

## Conclusions

Current improvements in embryo culture conditions are of major importance, but this may have been overvalued when compared to optimizing the environment where embryos should be placed and implanted, the so-called endometrium. An accurate, gentle, rapid, safe, real-time and especially low-cost procedure such as reproductive hysteroscopy might be proved a valuable diagnostic tool for a significant number of women who continue assisted reproduction treatments ignoring possible existing intrauterine pathology. Hysteroscopic examination of oocyte recipients has demonstrated a remarkably high rate of abnormalities of the intrauterine cavity, especially when considering the RIF-DOR group. Correction of these intrauterine lesions may improve the implantation rates and, subsequently, live birth rates.

## References

[1] Bakour SH, Jones SE, O'Donovan P (2006). Ambulatory hysteroscopy: evidence-based guide to diagnosis and therapy. Best Pract Res Clin Obstet Gynaecol.

[2] Cooper NA, Khan KS, Clark TJ (2010). Local anaesthesia for pain control during outpatient hysteroscopy: systematic review and meta-analysis. BMJ.

[3] Grimbizis GF, Tsolakidis D, Mikos T, Anagnostou E, Asimakopoulos E, Stamatopoulos P, Tarlatzis BC (2010). A prospective comparison of transvaginal ultrasound, saline infusion sonohysterography, and diagnostic hysteroscopy in the evaluation of endometrial pathology. Fertil Steril.

[4] Salazar CA, Isaacson KB (2018). Office operative hysteroscopy: an update. J Minim Invasive Gynecol.

[5] Gaskins AJ, Zhang Y, Chang J, Kissin DM (2023). Predicted probabilities of live birth following assisted reproductive technology using United States national surveillance data from 2016 to 2018. Am J Obstet Gynecol.

[6] Fiorentino F, Bono S, Biricik A (2014). Application of next-generation sequencing technology for comprehensive aneuploidy screening of blastocysts in clinical preimplantation genetic screening cycles. Hum Reprod.

[7] Taylor E, Gomel V (2008). The uterus and fertility. Fertil Steril.

[8] Kogan L, Dior U, Chill HH, Karavani G, Revel A, Shushan A, Simon A (2016). Operative hysteroscopy for treatment of intrauterine pathologies does not interfere with later endometrial development in patients undergoing in vitro fertilization. Arch Gynecol Obstet.

[9] Molina-García L, Hidalgo-Ruiz M, Cocera-Ruíz EM, Conde-Puertas E, Delgado-Rodríguez M, Martínez-Galiano JM (2019). The delay of motherhood: reasons, determinants, time used to achieve pregnancy, and maternal anxiety level. PLoS One.

[10] Coughlan C (2018). What to do when good-quality embryos repeatedly fail to implant. Best Pract Res Clin Obstet Gynaecol.

[11] Barri PN, Coroleu B, Clua E, Tur R, Boada M, Rodriguez I (2014). Investigations into implantation failure in oocyte-donation recipients. Reprod Biomed Online.

[12] Munro MG (2019). Uterine polyps, adenomyosis, leiomyomas, and endometrial receptivity. Fertil Steril.

[13] Riemma G, Vitale SG, Manchanda R (2022). The role of hysteroscopy in reproductive surgery: today and tomorrow. J Gynecol Obstet Hum Reprod.

[14] Apirakviriya C, Rungruxsirivorn T, Phupong V, Wisawasukmongchol W (2016). Diagnostic accuracy of 3D-transvaginal ultrasound in detecting uterine cavity abnormalities in infertile patients as compared with hysteroscopy. Eur J Obstet Gynecol Reprod Biol.

[15] Bosteels J, van Wessel S, Weyers S, Broekmans FJ, D'Hooghe TM, Bongers MY, Mol BW (2018). Hysteroscopy for treating subfertility associated with suspected major uterine cavity abnormalities. Cochrane Database Syst Rev.

[16] (2021). Guidance regarding gamete and embryo donation. Fertil Steril.

[17] Al-Turki HA (2018). Hysteroscopy as an investigation tool in recurrent implantation failure in vitro fertilization. Saudi Med J.

